# Dynamic profiles, biodistribution and integration evaluation after intramuscular/intravenous delivery of a novel therapeutic DNA vaccine encoding chicken type II collagen for rheumatoid arthritis in vaccinated normal rodent

**DOI:** 10.1186/s12951-019-0528-5

**Published:** 2019-09-06

**Authors:** Xiao Zhao, Juan Long, Fei Liang, Nan Liu, Yuying Sun, Yongzhi Xi

**Affiliations:** Department of Immunology and National Center for Biomedicine Analysis, the Fifth Medical Center (formerly known as Beijing 307 Hospital), Chinese PLA General Hospital, No. 8, Dongda Ave, Fengtai District, Beijing, 100071 People’s Republic of China

**Keywords:** Therapeutic DNA vaccine, Dynamic profiles, Biodistribution, Genomic integration, Rheumatoid arthritis

## Abstract

**Background:**

The persistence, biodistribution, and risk of integration into the host genome of any new therapeutic DNA vaccine must be established in preclinical studies. We previously developed the DNA vaccine pcDNA-*CCOL2A1* encoding chicken type II collagen (CCII) for the treatment of rheumatoid arthritis (RA). In the present study, we characterized its dynamic profile, biodistribution, and potential for genomic DNA integration in normal vaccinated rodent.

**Results:**

A real-time quantitative PCR analysis (RT-qPCR) of animals administered a single dose of pcDNA-*CCOL2A1* (300 μg/kg by intramuscular injection) showed that *CCOL2A1* mRNA level in the blood peaked between 2 and 6 h post-immunization and then rapidly declined, and was undetectable between day 1–42. *CCOL2A1* transcript was detected at the muscle injection site on days 3–14 post-immunization. Starting from day 14, the transcript was detected in the heart, liver, lung, and kidney but not in the spleen or thymus, and was expressed only in the lung on day 28. There was no *CCOL2A1* mRNA present in the testes or ovaries at any time point. Non-invasive in vivo fluorescence imaging revealed CCII protein expression from 2 h up to day 10 and from 2 h up to day 35 after administration of pcDNA-*CCOL2A1* via the intravenous and intramuscular routes, respectively; the protein had disappeared by day 42. Importantly, *CCOL2A1* was not integrated into the host genome.

**Conclusions:**

These results indicate that pcDNA-*CCOL2A1* vaccine is rapidly cleared within a short period of time and is therefore safe, and merits further development as a therapeutic vaccine for RA treatment.

## Background

The American College of Rheumatology and European League Against Rheumatism have been continuously updating the guidelines and recommendations for rheumatoid arthritis (RA) treatment over the last two decades due to the constant development of new anti-rheumatic drugs [1, 2]. Among them, DNA and selected autoantigen- or peptide-based vaccines are some of the most promising new therapeutic strategies for RA treatment [3–7]. The recently developed Rheumavax, which is based on autologous dendritic cells modified with a nuclear factor κB inhibitor exposed to four citrullinated peptide antigens, has completed safety trials with promising results in a single-center, open-labeled, first-in-human phase I trial [8]. Additionally, several DNA vaccines based on anti-cytokine and -chicken type II collagen (CCII) and targeting the B7-2/CD28 co-stimulatory signaling pathway have completed pre-clinical trials [3–7, 9–15]. By comparison, the advantage of DNA vaccines for the treatment of RA is obvious and fascinating. First, compared to immunosuppressants, such as synthetic and biological disease-modifying anti-rheumatic drugs (DMARDs) [16, 17] even newly marketed oral Janus kinase (JAK) inhibitors [18], DNA vaccines don’t induce generalized immunosuppression which is associated with various adverse effects and thus do not compromise host defense and increase risk of infection [3–7, 9–11, 14, 15]. Secondly, compared to the emerging antigen-specific immunotherapies involve tolerogenic antigens or peptides, dendritic cells (DCs) and regulatory T cells (Tregs) therapies, small interfering RNAs (siRNA)-based therapy, etc. [19–25], DNA vaccines can be prepared in large quantities and at low cost, and they can be stored for long periods due to their good stability [12, 13]. Lastly, more to the point is the fact that the antigen-specific DNA vaccines show good therapeutic efficacy and relative long-term durable responses potentially by inducing immune tolerance targeted specific self-antigens to regulate the whole network of immune system and re-establish immune balance [3–7, 9, 14, 15]. For these reasons, DNA vaccines will undoubtedly be an innovative personalized therapeutic strategies in the arena of RA immunotherapy.

Type II collagen (CII) is a critical autoantigen in RA pathogenesis. Native chicken type II collagen (nCCII) was shown to be effective in the treatment of RA patients [26–29]. However, the purified nCCII can cause its structural degeneration and loss of biological function, and can also be contaminated by pathogenic viruses. Based on these valuable findings, we previously developed the pcDNA-*CCOL2A1* therapeutic vaccine encoding CCII and found that its efficacy was comparable to that of the current gold standard drug methotrexate (MTX) in a rat model of collagen-induced arthritis (CIA) [9]. More recently, we showed that pcDNA-*CCOL2A1* was safe and well-tolerated as a therapeutic vaccine, did not markedly affect the balance of humoral and cellular immune responses, and had no immunogenicity in normal rats when administered by intramuscular injection [10, 11]. Integrated all research evidence from our studies strongly suggest that pcDNA-*CCOL2A1* is promising therapeutic DNA vaccine. However, it should be pointed out here that DNA vaccine pcDNA-*CCOL2A1* was injected intramuscularly for the targeted DNA delivery in the present study. Actually, there have so far been various vehicles or technologies for DNA delivery used in preclinical or clinical trials [30–32]. The viral vector is the earliest and most used carrier to deliver and protect DNA, but its use in gene therapy have been restricted due to the risks of triggering severe immune response and inserting into genome. Consequently, the non-viral DNA delivery systems such as physical methods and chemical vectors have also been developed to transfer DNA into cells. The physical methods do not use carriers but relies on a physical force that enhance the cell membrane permeability to facilitate the gene into cells, including needle, microneedle and jet injection, electroporation, gene gun, ultrasound, and hydrodynamic injection. The chemical vectors are to prepare the carriers by chemical methods to carry DNA into the nucleus, such as electrostatic attraction between anionic DNA and a cationic lipid or polymer, DNA encapsulation using various biodegradable polymers to prepare micrometric or nanometric spherical structures containing DNA by various advanced preparation technologies, and biological transfection mediators for example protein transduction domains (PTDs) namely cell penetrating peptides and exosomes, etc. [31, 32]. All of these technologies will support the development of preparations and formulations for the clinical application of this DNA vaccine pcDNA-*CCOL2A1* in the near future.

For any new therapeutic DNA vaccine with high druggability, whether exogenous genes can be quickly removed and unintegrated into the host genome after taking effect in vivo is not only a very important scientific issue but also is related to whether the new vaccine can be approved to be formally applied to clinical practice. According to the vaccine regulations by both FDA and CFDA, the study on pharmacokinetics of any new developed DNA vaccines must be carried out before clinical trial application. These include the evaluation of dynamic profiles or persistence, biodistribution, integration risk, etc. [33–35]. To this end, in the present study we evaluated the dynamic profiles of pcDNA-*CCOL2A1* in blood and the biodistribution of *CCOL2A1* mRNA and CCII protein, and investigated whether plasmid integration occurred in various tissues of vaccinated normal rodent. This is the first time to investigate the pharmacokinetics of this DNA vaccine from both gene and protein level. The revealing of pharmacokinetics and genome integration risk will be very helpful for future clinical trial applications.

## Results

### Persistence of *CCOL2A1* gene in peripheral blood from vaccinated normal rats

We recently reported that vaccination with pcDNA-*CCOL2A1* at a dose of 3 mg/kg was well-tolerated by rats [11]. The optimal therapeutic dose of 300 μg/kg did not induce production of anti-CII IgG and did not alter the expression levels of most cytokines and T-lymphocyte subsets in normal rats [10], suggesting that pcDNA-*CCOL2A1* is a promising therapeutic DNA vaccine with high druggability. We therefore evaluated the dynamic profiles of pcDNA-*CCOL2A1* in rats at the same dose by assessing clearance after vaccination.

**Fig. 1:**
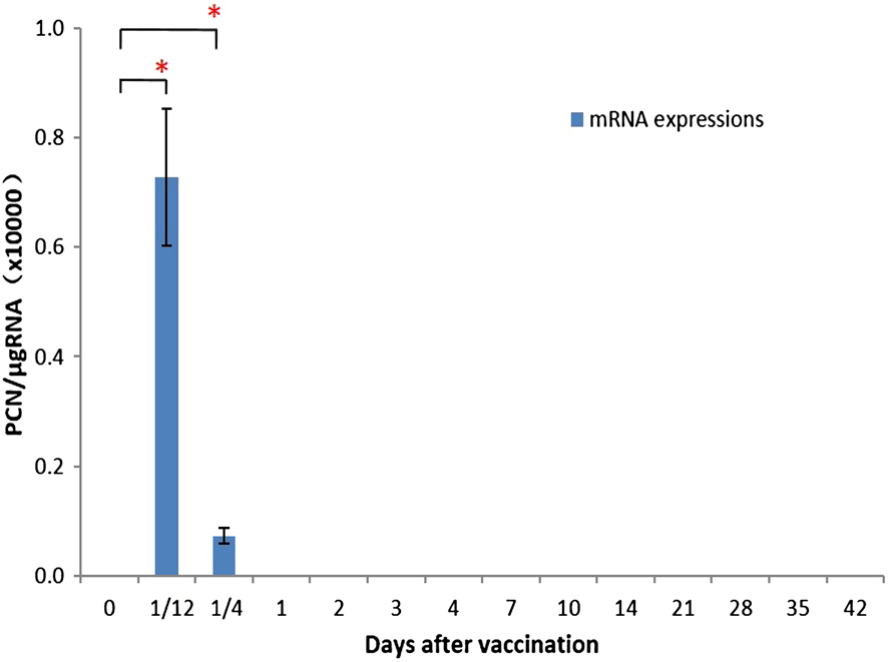
Persistence of *CCOL2A1* gene in peripheral blood from vaccinated normal rats. pcDNA-*CCOL2A1* was intramuscularly injected into normal Wistar rats with a single dose of 300 μg/kg (N = 6). Peripheral blood samples were continuously harvested at various time points and processed for qPCR determination. Data are expressed as PCN/μg RNA; LOD = 200 PCNs/μg RNA and LLOQ = 1000 PCNs/μg RNA. “PCNs” represent “plasmid copy numbers”. *P < 0.05 compared with the value of 0 d using the *t*-test

We analyzed the persistence of pcDNA-*CCOL2A1* in peripheral blood from vaccinated normal rats after a single intramuscular injection by measuring *CCOL2A1* mRNA levels from six rats (three of each sex) by RT-qPCR at 0, 2 and 6 h and on days 1, 2, 3, 4, 7, 10, 14, 21, 28, 35, and 42 after vaccination. Copies of plasmid DNA were detectable at 2 h post-injection (Fig. 1); at 6 h, plasmid DNA was still present in the blood of most animals, although the plasmid copy number (PCN) was reduced compared to the 2-h time point. Rapid clearance from the blood was subsequently observed: on day 1 post-injection, the plasmid was almost undetectable except in a few animals that showed negligible expression: mean PCN/μg RNA values on day 1 (= 265) and day 3 (= 205) were between the lower limit of quantitation (LLOQ) and limit of detection (LOD), and values at all other time points were below the LOD. In addition, no signal was detected before vaccination (0 h).

### Biodistribution of *CCOL2A1* gene in tissues of vaccinated normal rats

**Fig. 2:**
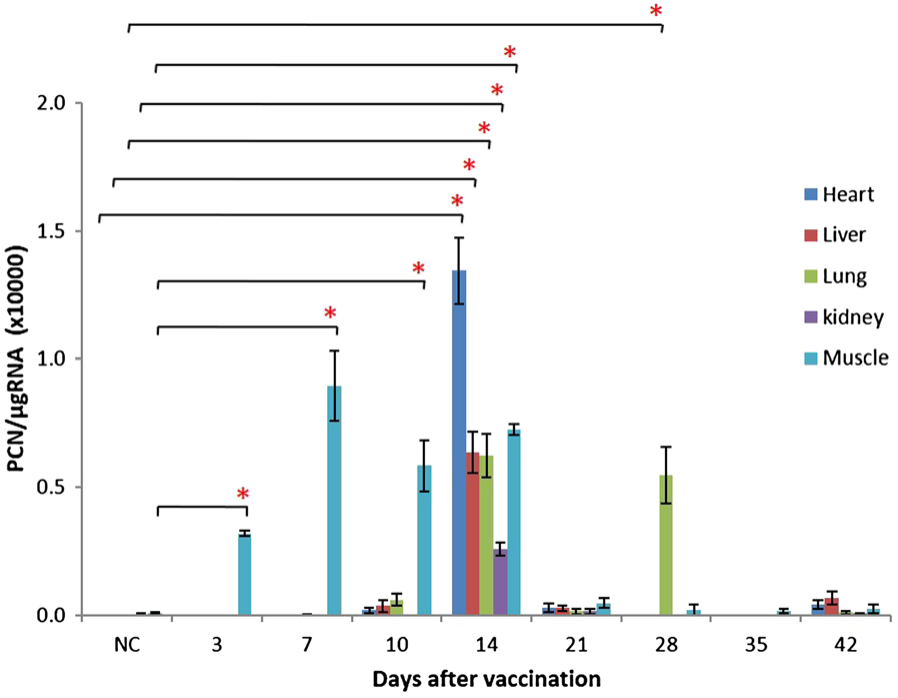
Biodistribution of *CCOL2A1* gene in various tissues from vaccinated normal rats. pcDNA-*CCOL2A1* was intramuscularly injected into normal Wistar rats with a single dose of 300 μg/kg (N = 6). On days 3, 7, 10, 14, 21, 28, 35 and 42 after vaccination, a panel of 10 tissues/organs were harvested and processed for qPCR determination. Data are expressed as PCN/μg RNA; LOD = 200 PCNs/μg RNA and LLOQ = 1000 PCNs/μg RNA. For graphing purposes, values from spleen, thymus, ovary, testis and blood are not shown which are less than the LOD or above the LOD but below the LLOQ. *P < 0.05 comparing values of various time-points after vaccination vs. the one of NC using the *t*-test. “NC” represent “normal control”

We next investigated the biodistribution of pcDNA-*CCOL2A1* in various tissues of vaccinated normal rats after a single intramuscular injection. *CCOL2A1* mRNA expression was assessed by RT-qPCR on days 3, 7, 10, 14, 21, 28, 35, and 42 after vaccination, respectively. Between days 3 and 14, *CCOL2A1* transcript was detected at the muscle injection site in most animals, but disappeared thereafter. On day 14, *CCOL2A1* mRNA was detected in the heart, liver, lung, and kidney but not in the spleen or thymus. However, by day 28, the transcript was present only in the lungs and not in other tissues. There was no product above the LLOQ detected before day 14 or on days 21, 35, or 42 after vaccination in any tissue (Fig. 2). In addition, *CCOL2A1* mRNA was not detected in the peripheral blood from days 3 through 42 after vaccination (data not shown), which was consistent with results obtained from continuously harvested blood samples. The transcript was not detected at any time point in the male or female gonads (data not shown). As expected, there was no signal above the LOD in the normal saline (NS)-treated negative control and reaction sentinel samples, indicating that no cross-contamination occurred during PCR analysis.

### Biodistribution and persistence of CCII protein in vaccinated normal rodent

**Fig. 3:**
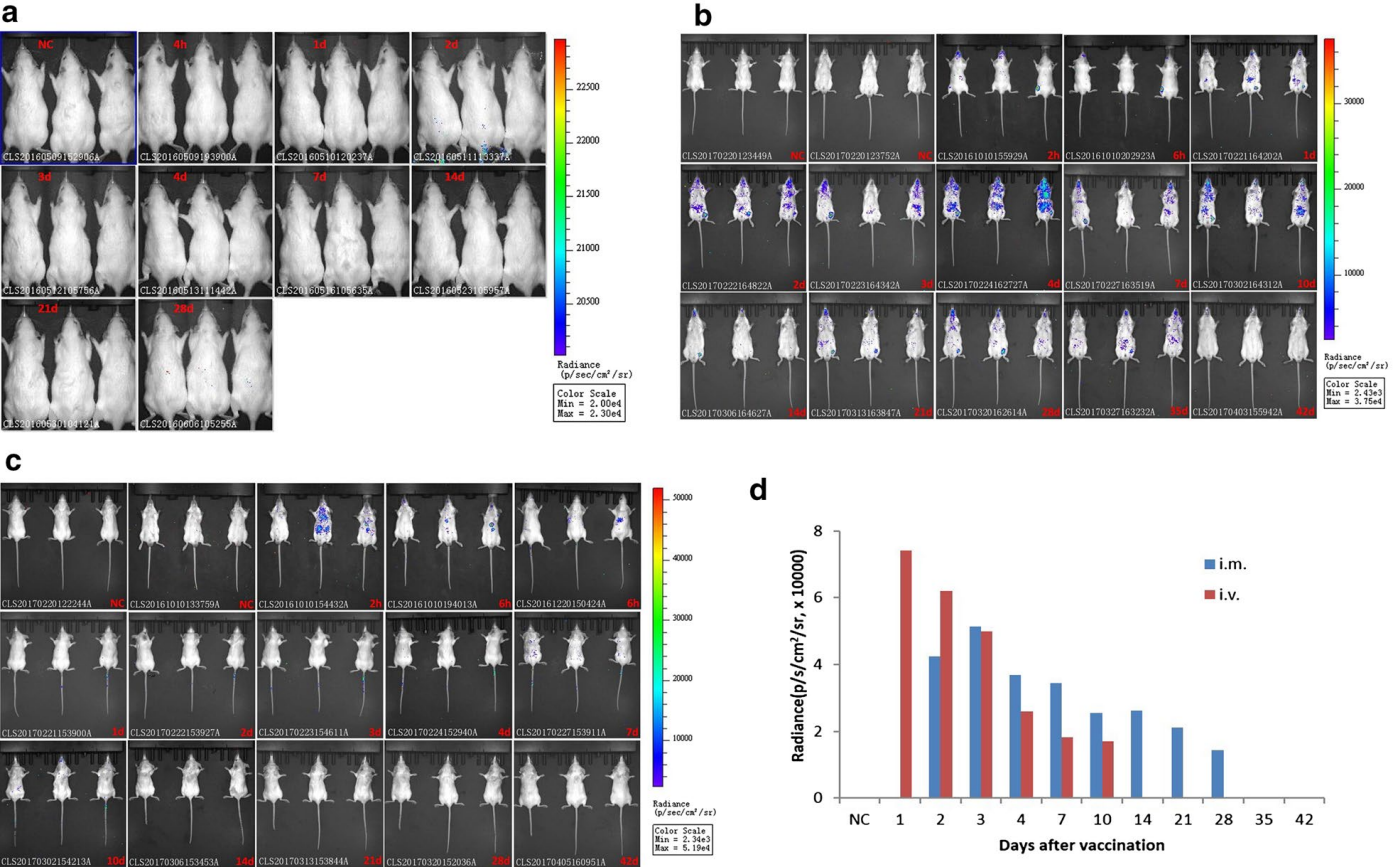
Identification of biodistribution and persistence of CCII protein in vaccinated normal rodent at different time points after vaccination with Luciferase gene-labeled pcDNA-*CCOL2A1*. **a** BLI images of Luciferase gene-labeled pcDNA-*CCOL2A1* in vaccinated normal Wistar rats by intramuscular injection (three female rats as the representatives). **b** BLI images of Luciferase gene-labeled pcDNA-*CCOL2A1* in vaccinated normal BALB/c mice by intramuscular injection (three males are shown here). **c** BLI images of Luciferase gene-labeled pcDNA-*CCOL2A1* in vaccinated normal BALB/c mice by intravenous injection (the dorsal images are from three females, and the abdominal ones from three males). **d** Quantification of the more continuous BLI signal in vaccinated normal BALB/c mice which data are from male mouse 1# in intramuscular injected muscle site and female mouse 3# in intravenous injected tail site, respectively. The BLI images were from 3 independent experiments

To establish the dynamic profiles of CCII protein in vaccinated normal rats, we carried out in vivo bioluminescence imaging (BLI) after intramuscular injection of 300 μg/kg luciferase-conjugated pcDNA-*CCOL2A1*. There was almost no bioluminescence in the back and abdomen of rats at 4 h and on days 1, 2, 3, 4, 7, 14, 21, and 28 post injection in all three repeated and independent experiments (Fig. 3a). Considering that the dosage may be too low to generate protein, a higher dosage of 3 mg/kg DNA vaccine was injected intramuscularly in rats including the ones shaved. Likewise, there is still no observable BLI signal appearing (data not shown). Given the weakness of the signal due to the thickness of the fur, we also investigated the dynamic profiles of CCII protein in BALB/c mice injected intramuscularly or intravenously with the vaccine. The signal intensity was significantly higher in mice than in rats at comparable doses of pcDNA-*CCOL2A1* (430 μg/kg). The Fig. 3b showed the results of biodistribution and persistence of CCII protein from three vaccinated MALE mice injected via the intramuscular route. The BLI signals appeared in the injected left hind thigh site starting from 2 h post-injection until day 35. The results from three female mice were not displayed because there were almost no bioluminescence observed in the injected left hind thigh site except abdominal image on day 1 and 2 post-injection. The Fig. 3c showed the dorsal imaging results of CCII protein from three vaccinated FEMALE mice from 6 h to day 42 (signals appeared in the injected tail site from 6 h to day 10) and the abdominal image from three vaccinated MALE mice only at 2 h and 6 h (signals may appeared in the liver) injected via the intravenous route. The signals from tail injection site were weaker and only appeared on male mouse 1# from day 1 to 7; but the signals may from liver were never observed on FEMALE mice in 3 independent experiments. The BLI values each day in Fig. 3d were from one mouse which showed the more continuous expression of CCII protein in injected muscle or tail site, respectively. And the BLI signal intensity varied by 10^4^ fold. In short, the CCII protein had disappeared by day 42 both vaccination via the intramuscular and intravenous route in mice. These results indicate that in vivo imaging using a combination of pcDNA-*CCOL2A1* and the luciferase gene can profile the relative biodistribution and persistence of CCII protein in the host.

### Genomic DNA integration of the *CCOL2A1* gene in vaccinated normal rats

To assess the risk of integration of the *CCOL2A1* gene into the host genome, we performed RT-qPCR analysis on day 42 of blood and various tissue samples from normal rats intramuscularly injected with a single dose of pcDNA-*CCOL2A1* or NS. The internal control gene (*β*-*actin*) was detected in each DNA preparation. *CCOL2A1* was not detected in blood samples from NS-injected rats, with PCN/μg DNA below the LOD (Table 1). Moreover, *CCOL2A1* was not detected in the majority of vaccinated normal rats, and the PCN was extremely low (below the LOD) in the few positive samples. Importantly, testis and ovary samples from all rats were negative for the *CCOL2A1* gene. These results indicate that the *CCOL2A1* gene was not integrated into the genomic DNA of vaccinated rats.

**Table 1:** The identification of genomic integration of *CCOL2A1* gene in vaccinated normal rats on day 42 after vaccination following single intramuscular injection (N = 6)

Tissues	Mean ± SD (PCN/µg DNA)	
	NS negative control	Vaccinated group
Heart	UD	UD
Liver	UD	UD
Spleen	UD	UD
Lung	UD	UD
Kidney	UD	UD
Thymus	UD	UD
Muscle	UD	98.65 ± 70.49
Ovary	UD	UD
Testis	UD	UD
Blood	157.9 ± 105.1	91.63 ± 45.05

Values ≤ LOD (182 PCN/µg DNA) indicate a negligible risk of *CCOL2A1* gene integration. “UD” respresent “undetermined”. A panel of 10 tissues/organs were harvested and processed for qPCR determination. These data are representative of three experiments. Three separate experiments yielded similar results

## Discussion

Therapeutic DNA vaccines have considerable advantages over traditional DMARDs, biological agents, tofacitinib, and glucocorticoids, since they are not only capable of inducing cellular and humoral immune responses but can also modulate the immune system. In fact, the critical aspect of therapeutic DNA vaccines is induction of immune tolerance [3–7, 36, 37], which is especially important for the treatment of refractory autoimmune diseases such as RA, multiple sclerosis, and insulin-dependent diabetes mellitus that involve dysfunctional innate and adaptive immune systems. However, there is always a concern regarding the safety of therapeutic DNA vaccines due to a lack of long-term safety data in humans. The results of our pre-clinical assessment indicate that *CCOL2A1* transcript and protein expressed from pcDNA-*CCOL2A1* were rapidly cleared in injected normal rats and that the *CCOL2A1* gene was not integrated into the host genome, providing evidence for the safety of this vaccine.

In this study, we monitored the biodistribution of *CCOL2A1* mRNA in rats administered a single injection of 300 μg/kg pcDNA-*CCOL2A1* and found that *CCOL2A1* transcript level in peripheral blood peaked between 2 and 6 h post-immunization and then rapidly decreased, and was undetectable on days 1–42. An analysis of various tissues revealed that the transcript was only present at the injection site–the muscle between days 3 and 14 post-immunization, after which time it was detected in heart, liver, lung, and kidney but not in the spleen or thymus. On day 28, the expression was restricted to the lung. Importantly, there was no *CCOL2A1* transcript present in the reproductive organs at any time point. These results confirm the rapid clearance of the pcDNA-*CCOL2A1* and underscore its safety advantage over vaccines derived from pathogenic microorganisms that may persistently express an exogenous gene in the host. Our results are mostly consistent with data from other animal species demonstrating that plasmid DNA introduced by injection or electroporation can be cleared within several minutes, hours, or up to 60 days [38–40]. Peak times of maximum plasmid DNA concentration at the injection site showed similar variability. In previous studies, the biodistribution of naked plasmid DNA encoding fibroblast growth factor type 1 was limited to the injection site while plasma and serum were negative in the majority of treated patients, which differed from results obtained using vascular endothelial growth factor-A vaccine [41–43] and the pcDNA-*CCOL2A1* therapeutic vaccine used here. These discrepancies are likely due to the target of different genes and the use of different expression vectors, although in all of these studies a human or animal structural gene was targeted.

The biodistribution and persistence of DNA vaccines are influenced by the type of expression vector used (high vs. low expression) as well as the route of administration (e.g., intramuscular, intravascular, tail vein injection, particle bombardment, and in vivo electroporation). However, most of the existing research has focused on exogenous genes from pathogenic microorganisms such as human immunodeficiency virus, hepatitis C virus, malaria, measles, and Ebola rather than those from humans or other animals [38–40, 44–47]. In this study we used the pcDNA3.1(+) expression vector, which is highly stable and has a high copy number, and is therefore widely used for transient gene expression. For convenience in future clinical applications, we chose to deliver the vaccine by intramuscular injection. Although other routes of administration can in theory increase the transfection efficiency, the persistence of plasmid DNA in the target tissue may be affected, which could increase the risk of integration into the host genome. It is also possible that the dynamic profiles and biodistribution of DNA vaccines depend on the target gene and expression vector that is used, although this remains to be confirmed by more detailed analyses.

Molecular imaging with reporter genes can be useful for non-invasive monitoring of exogenous and endogenous target gene expression and intracellular biological events [48, 49]. In the present study, we used this approach to investigate the therapeutic action of pcDNA-*CCOL2A1* in vivo by evaluating the distribution of CCII protein in real time. We used a luciferase reporter to track the expressed CCII protein. Under conditions of ATP and oxygen, the substrate luciferin is oxidized by the expressed luciferase, and the resultant bioluminescence enabled quantitation and localization of the fused *CCOL2A1* gene product while avoiding background fluorescence from fur or from food in the gastrointestinal tract. Thus, BLI can be used to detect expressed proteins with high sensitivity, although the intensity of the signal is weaker than that of fluorescent molecules. For this reason, it was difficult to detect signals in vaccinated Wistar rats. In live imaging experiments of vaccinated mice, there was considerable inter-individual variability. For example, not every mouse had a BLI signal at a given time point; moreover, differences in the signal were observed between the two sexes and between dorsal and abdominal positions of signal acquisition. Besides these inherent factors, the injection speed and site may have contributed to the variability. Therefore, we have presented only relatively consistent results from a few mice.

Plasmid DNA vectors can potentially be incorporated into the host genome via random integration, homologous recombination, or a retroviral mechanism [31]. This can increase the probability of chromosomal insertion mutation, genomic instability, dysregulation of cell growth, disruption/inactivation of tumor suppressor genes, and oncogenesis [30, 50, 51]. Integration of exogenous DNA into germ cells can induce genetic defects in offspring [52]. Although random integration of plasmid DNA into the host genome following intramuscular delivery or electroporation has been reported [53], the probability of such events is considered to be extremely low [44, 45, 54–57]. Nonetheless, we investigated the potential for genomic integration of pcDNA-*CCOL2A1* and found that no *CCOL2A1* transcript was detected in any of the analyzed tissues at a detection limit of approximately 182 copies/μg DNA, indicating that the risk of integration of plasmid DNA vaccines delivered by intramuscular injection is negligible. The mammalian genome is thought to have a mechanism for identifying and degrading foreign nucleotide sequences [58, 59]; our observations support this possibility [46, 54]. It is worth noting that tissues that were positive for *CCOL2A1* transcript were found to be negative in the analysis of plasmid DNA integration. We can therefore conclude that the plasmid DNA remained extrachromosomal and was rapidly degraded, and therefore posed little risk of germline transmission. So far there are several consensus mechanisms by which plasmid DNA vaccines including our pcDNA-*CCOL2A1* are cleared from the vaccinated animals or human bodies though we did not do this experiment in the present study. First mechanism is that extracellular and blood plasmid DNA is quickly degraded by enzymes such as nuclease [3, 36]. Second mechanism is that antigen-expressing cells from muscle can be cleared by immune system cells [3, 36]. Third mechanism is that plasmid DNA vaccines are degraded in cytoplasm and then released through exocytosis [60]. Fourth mechanism is that plasmid DNA vaccines by intravascular delivery are quickly degraded in liver [36]. In addition, plasmid DNA vaccines in gene gun bombarded skin are quickly cleared due to the normal sloughing of the epidermis [60].

## Conclusions

In summary, pcDNA-*CCOL2A1* was rapidly cleared from most tissues in rats within about 4–6 weeks of intramuscular administration at a dose of 300 μg/kg. Importantly, the exogenous *CCOL2A1* gene was not integrated into the host genome. These results strongly support the further clinical development of pcDNA-*CCOL2A1* as a therapeutic vaccine for RA treatment.

## Methods

### pcDNA-*CCOL2A1* vaccine

The pcDNA-*CCOL2A1* vaccine was generated from the eukaryotic pcDNA-*CCOL2A1* expression vector previously constructed in our laboratory [9] (GenBank databases accession Nos. AY046949 and AF452711) and contained a 4837-bp full-length cDNA encoding CCII with deleted N-propeptide, signal peptide sequence, and Kozak consensus sequence [61]. The resultant recombinant plasmid pcDNA-*CCOL2A1* was produced in *Escherichia coli* and purified using the Endo-free Mega-prep kit (Qiagen, Valencia, CA, USA).

The luciferase labeled pcDNA-*CCOL2A1* vaccine was constructed by restriction enzyme *Hin*dIII and protect bases, and contained a Kozak consensus sequence and a flexible peptide sequences at the 5′, 3′-luciferase gene, respectively. The luciferase and *CCOL2A1* genes were linked by flexible peptide sequences.

### Animals

Inbred female/male Wistar rats (4–6 weeks old) and BALB/c mice (4–5 weeks old) were obtained from the Animal Breeding Center of the Academy of Military Medical Sciences (Beijing, China) and maintained under specific pathogen-free conditions. Experiments were performed according to the guidelines of the Academy of Military Medical Sciences Animal Welfare Committee.

### Vaccination with pcDNA-*CCOL2A1*

A total of 54 normal rats were used in this study; 48 (including males and females) received a single intramuscular injection of 300 μg/kg pcDNA-*CCOL2A1* in the left hind limb musculi biceps femoris. Six rats per time point (three of each sex) were sacrificed at different time points after vaccination. The rats were anesthetized with ether, and various tissues were harvested for analysis of tissue distribution and DNA integration. The remaining six rats were injected with normal saline (NS) as a negative control.

To evaluate the persistence of pcDNA-*CCOL2A1*, six rats (three of each sex) were intramuscularly injected in the left hind limb musculi biceps femoris with a single 300 μg/kg dose of pcDNA-*CCOL2A1*. Blood samples were collected from the orbital venous plexus at different time points post-vaccination for RNA extraction.

### RT-qPCR analysis of *CCOL2A1* mRNA biodistribution

Samples of blood and tissue including heart, liver, spleen, lung, kidney, thymus, muscle (injection site), and ovary or testis were obtained at various time points and total RNA was extracted using TRIzol reagent (Life Technologies, Carlsbad, CA, USA) according to the manufacturer’s protocols. The RNA was extracted with chloroform, precipitated with isopropyl alcohol, and dissolved in diethyl pyrocarbonate-treated water, and 1 μg was reverse transcribed in a 20-μl reaction volume using the reverse transcription reagent (Life Technologies) at 25 °C for 5 min followed by 50 °C for 60 min and 70 °C for 15 min before incubation on ice. RT-qPCR was carried out using Platinum Taq DNA polymerase (Life Technologies) with 1 μl of reverse-transcribed product as the template and TaqMan probe as the detection reagent. The resultant cDNA was amplified in a 7500 Real-Time PCR system (Applied Biosystems, Foster City, CA, USA) under the following cycling conditions: 95 °C for 2 min, and 40 cycles of 95 °C for 10 s and 60 °C for 30 s. Rat *β*-*actin* was used as an internal control. The amplified fragment size and primer and probe sequences of *CCOL2A1* and *β*-*actin* are shown in Table 2. RNA samples isolated from different tissues were analyzed in parallel with negative control samples (from rats injected with NS) and reaction sentinel control samples (using ddH2O as the template). A positive control consisting of pcDNA-*CCOL2A1* combined with cDNA from blood and various tissues from rats treated with NS was used as a template in each set of PCR reactions.

**Table 2:** The amplified fragment size, primer and probe sequences of *CCOL2A1* and *β*-*actin* by quantitative polymerase chain reaction

Gene	Primer/probe name	Prime/probe sequence	Fragment size (bp)
Rat *β*-*actin*	Rat *β*-*actin* F-primer	5′-CTCATGCCATCCTGCGTCT-3′	116
	Rat *β*-*actin* R-primer	5′-ACGCACGATTTCCCTCTCA-3′	
	Rat *β*-*actin* probe	5′-TGGCCGGGACCTGACAGACTACC-3′	
*CCOL2A1*	*CCOL2A1* F-primer	5′-TCTTGTTGGTCCCAGAGGTGA-3′	118
	*CCOL2A1* R-primer	5′-ACCCTTGGGTCCGTCAGTG-3′	
	*CCOL2A1* probe	5′-CGTGGATTCCCCGGTGAACGC-3′	

DNA standards (pcDNA-*CCOL2A1* and the reference gene *β*-*actin* serially diluted in blood and tissue extracts from vehicle-treated rats) were run in parallel; the quantifiable range was from 10 to 1 × 10^7^ for pcDNA-*CCOL2A1* and 1 × 10^2^ to 1 × 10^8^ for *β*-*actin* plasmid copy numbers (PCNs). The LOD and the LLOQ per reaction were 10 and 50 PCNs, respectively (that is, 200 and 1000 PCNs/μg RNA). Acceptability criteria for runs included standard curve correlation coefficients ≥ 0.998 as well as PCN values below the LOD for both the reagent controls (reactions lacking template) and extraction negative controls (reactions using NS/vehicle-treated rat cDNA). Specimen acceptability criteria included a threshold cycle (Ct; i.e., the cycle at which the detected reporter signal was above baseline fluorescence) difference between sample duplicates of ≤ 0.5 (for samples above the LLOQ). To quantify plasmid and *β*-*actin* transcript levels, a standard curve was generated by plotting the mean Ct value of each standard against the logN of the starting copy number obtained by RT-qPCR. Sample values were then fitted to the curve by linear regression analysis; the arithmetic mean copy number of three duplicate reactions was calculated, and data are expressed as PCN per μg RNA.

### In vivo BLI of CCII protein biodistribution

Six Wistar rats and BALB/c mice (three of each sex) were administered luciferase gene-labeled pcDNA-*CCOL2A1* via a single intramuscular injection at a dose of 300 μg/kg and 430 μg/kg, respectively. Another six BALB/c mice (three of each sex) were injected intravenously. Before imaging, immunized rats/mice were intraperitoneally injected with a single dose of 150 mg/kg d-luciferin substrate in NS. The animals were scanned at 2 and 6 h and on days 1, 2, 3, 4, 7, 10, 14, 21, 28, 35, and 42 after immunization under 2% (v/v) isoflurane-O_2_ anesthesia. BLI was performed using a dedicated small animal IVIS Spectrum Living Image System (PerkinElmer, Waltham, MA, USA) at 10–30 min post-luciferin administration. Data were analyzed using Living Image v.4.5 software (PerkinElmer). Bioluminescence signals were recorded as average radiance with units of photons/s/cm^2^/sr.

### Genomic DNA integration studies

Blood and other tissue samples including heart, liver, spleen, lung, kidney, thymus, muscle (injection site), and ovary or testis were analyzed to evaluate the extent of pcDNA-*CCOL2A1* integration into the host genome. Briefly, genomic DNA was extracted using the QIAamp DNA Mini kit (Qiagen, Hilden, Germany), and the purity was determined by assuring an absorption ratio at 260/280 nm of 1.8–2.0. Isolated genomic DNA was analyzed for the presence of the housekeeping gene (*β*-*actin*) and the exogenous *CCOL2A1* gene sequence by RT-qPCR. Briefly, 55 ng purified genomic DNA template was amplified using Platinum Taq DNA polymerase and TaqMan probe as well as sense and antisense primers targeting a 118-bp fragment of the *CCOL2A1* gene. The rat *β*-*actin*-encoding gene was amplified as an internal control (Table 2). Genomic DNA spiked with pcDNA-*CCOL2A1* was used as a positive control, and purified genomic DNA from NS-injected rats or ddH2O was used as a template in the negative control reactions. The amplification conditions were the same as those described in Section “RT-qPCR analysis of *CCOL2A1* mRNA biodistribution”.

**Fig. 4:**
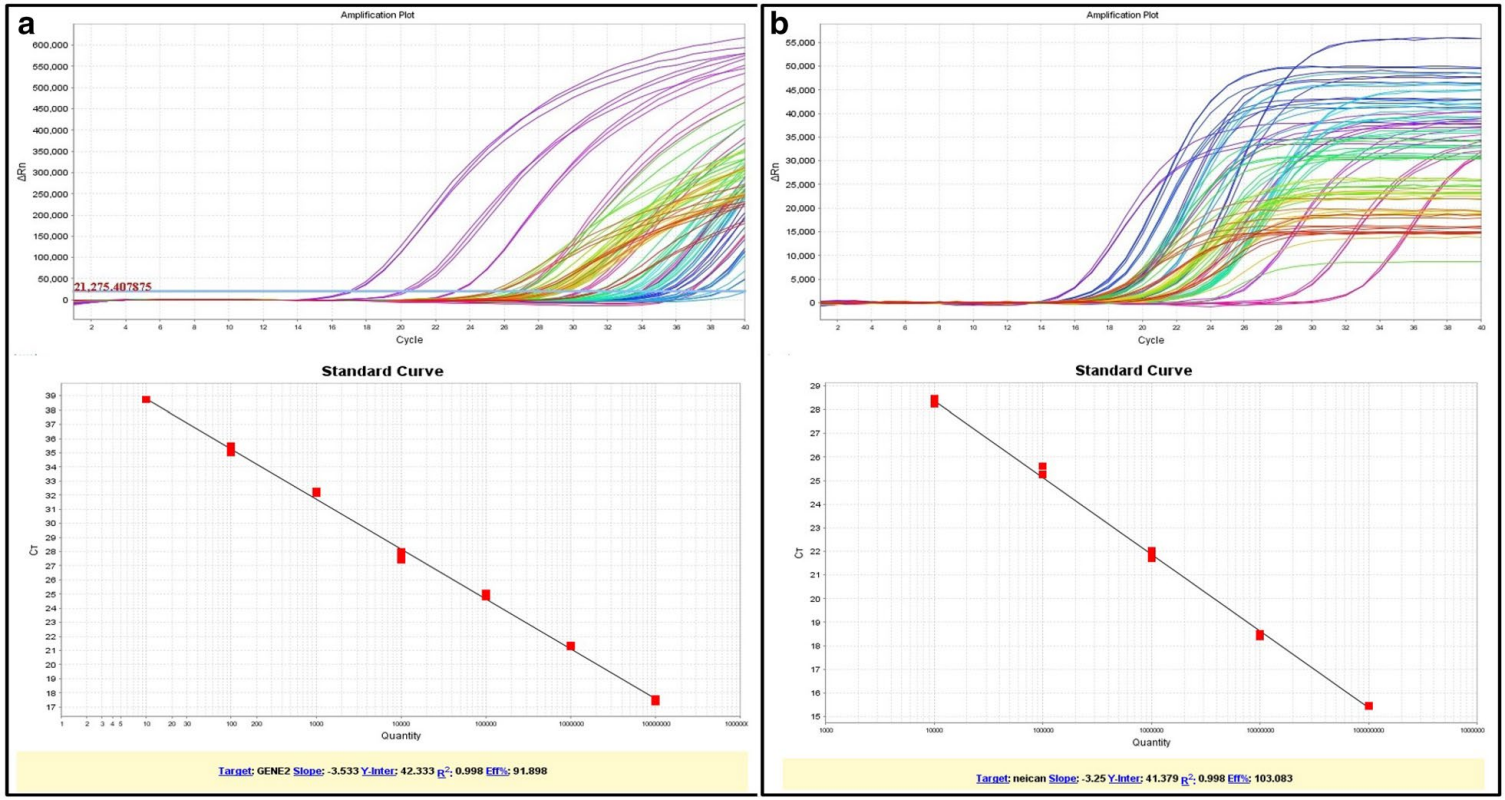
The representative amplification plots and linear standard curves of *CCOL2A1* and *β*-*actin* genes by RT-qPCR. **a** The amplification plots and linear standard curve of *CCOL2A1* gene; **b** The amplification plots and linear standard curve of *β*-*actin* gene. The upper plots in both **a** and **b** show the representative amplification curves including gene positive standards (purple lines) and samples to be tested (other colored lines) with three duplicates per sample in a 96-well plate by RT-qPCR. The X-axis is threshold cycle (Ct), the cycle at which the detected reporter signal was above baseline fluorescence; and Y-axis is ΔRn, the difference of fluorescence between the sample and baseline. The under plots are the representative linear standard curves from pcDNA-*CCOL2A1* positive control and *β*-*actin* internal control, respectively

DNA standards were run in parallel on each 96-well plate; the quantifiable range was from 10 to 1 × 10^7^ PCNs for pcDNA-*CCOL2A1*, and from 1 × 10^4^ to 1 × 10^8^ PCNs for *β*-*actin*. The LOD and LLOQ for each reaction were 10 and 50 PCNs, respectively (i.e., about 182 and 909 PCNs/μg DNA). The arithmetic mean copy number of duplicate reactions of each gene fragment in each sample were calculated and data are shown as described in Section “RT-qPCR analysis of *CCOL2A1* mRNA biodistribution”. The representative amplification plots and linear standard curves of *CCOL2A1* and *β*-*actin* genes were shown in Fig. 4a, b, respectively.

### Statistical analysis

Data derived from RT-qPCR are presented as mean ± SD and were analyzed with the Student’s *t* test using SPSS v.13.0 software (SPSS Inc., Chicago, IL, USA). *P *< 0.05 was considered statistically significant.

## Data Availability

All data generated or analyzed during this study are included in this published article.

## Abbreviations

*CCOL2A1*: chicken type II procollagen gene
CCII: chicken type II collagen
RA: rheumatoid arthritis
RT-qPCR: real-time quantitative PCR
CII: type II collagen
nCCII: native chicken type II collagen
MTX: methotrexate
CIA: collagen-induced arthritis
PCN: plasmid copy number
PCNs: plasmid copy numbers
LLOQ: lower limit of quantitation
LOD: limit of detection
NS: normal saline
BLI: bioluminescence imaging
Ct: threshold cycle
